# Journal Citation Reports

**DOI:** 10.5195/jmla.2019.646

**Published:** 2019-04-01

**Authors:** Anna Krampl

**Affiliations:** Library Assistant Professor, Head of Public Services, and Library Liaison to Surgery, Obstetrics and Gynecology, and Psychiatry, Skelton Medical Library, Mercer University School of Medicine, Macon, GA, krampl_a@mercer.edu

## INTRODUCTION

The need to evaluate and compare medical journals has become an ongoing struggle. Researchers and librarians have several citation and journal metric options that can help make assessments at the journal, article, and author level.

While the over-reliance on any journal-level metric has its shortcomings, the impact factor, also known as journal impact factor, remains one of the most widely used indicators of journal quality. Eugene Garfield first thought of the idea in 1955, but it would be decades later when the *Journal Citation Report* was first utilized in the 1970s [1]. The *Journal Citation Report* is one of the only sources of citation data on journals covering the areas of science and medicine, technology, and social sciences.

This resource review provides a general overview of the content and features of Clarivate Analytics’ Journal Citation Reports (JCR). It explains impact factors and journal rankings, and how they are calculated and can be utilized.

## CONTENT

An impact factor is a measure of the frequency with which the average article in a journal has been cited in a particular year. The higher a journal’s impact factor, the more frequently articles in that journal are cited. In this way, an impact factor can give an approximate indication of how prestigious or influential a journal is in its field. A journal’s impact factor is *not* a direct link to factors such as quality of peer review or quality of the content of a journals’ articles.

Impact factors for journals are sourced from several indexes in the Web of Science Core Collection published by Clarivate Analytics, such as the Science Citation Index and the Social Sciences Citation Index. As of November 2018, JCR has aggregated data from over 11,500 titles and over 230 disciplines.

To be covered in Web of Science and, therefore, have a chance to be given an impact factor, journals undergo a selection process and maintenance review. More information about this process can be found on the JCR website [2]. Clarivate Analytics is not a publisher of journals, which affords the resource a sense of objectivity. This sense of objectivity is perhaps more difficult to maintain for creators of metrics who are publishers of journals.

Essentially, a journal’s impact factor is derived by calculating the average number of times that articles in a journal are referenced by other journals within a future period of time. Calculating the JCR impact factor uses a ratio where the numerator is the number of times an article that was published in a specific journal in the previous two years was cited. The denominator is the number of total possible citable items published in that journal (note: JCR does not include items like letters and other editorial items).

Here is an example of a fictitious journal: *The Example Journal*. For the year 2017, Clarivate scans through a set of journals covered by JCR (11,500 titles as of November 2018). It counts the number of times that articles published in 2017 made reference to works published in *The Example Journal* in 2015 and 2016. If this number is 200, that is the numerator. Then it counts the total number of articles published by *The Example Journal* in 2015 and 2016. If this is 45, that is the denominator. Divide the 2 numbers to calculate the impact factor.

$$\frac{\begin{array}{c} \#\;\text{of references in }2017\;\text{to articles published}\\ \text{in }2015\;\text{and }2016\;\text{in }The\;Example\;Journal \end{array}}{\begin{array}{c} \#\;\text{of articles published in }2015\;\text{and }2016\\ \text{in }The\;Example\;Journal \end{array}}$$

The 2017 impact factor (IF) for *The Example Journal* is 4.444. Librarians and researchers are often asked what this number means. Is this a high impact factor? What is a good impact factor?

One important thing to keep in mind is the high variation in average impact factors across different subject areas. Practically speaking, it is rarely useful to compare journals from different subject areas. Hence, JCR classifies journals into a number of subject categories. For instance, the #1 ranked journal in the category of “Immunology” is *Nature Reviews Immunology*, with a 2017 impact factor of 41.982. The top ranked journal in “Economics” is the *Quarterly Journal of Economics* with a 2017 impact factor of 7.863. This should not be taken to mean that *Nature Reviews Immunolo*gy is a higher quality journal than the *Quarterly Journal of Economics*. Certain disciplines lend themselves to having articles cited at higher levels than others. They are both ranked #1 in their respective categories, and this should be the key takeaway.

**Figure 1:**
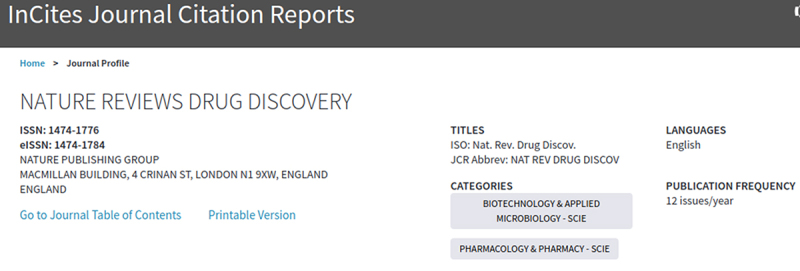
Journal Citation Reports (JCR) information for *Nature Reviews Drug Discovery*

Each journal in JCR is assigned to at least one subject category. In some cases, a journal may be assigned to more than one subject category; therefore, when comparing journals across related categories, it is possible to see the same journal title in different categories. In Figure 1, *Nature Reviews Drug Discovery* is listed in two categories: “Biotechnology & Applied Microbiology” and “Pharmacology & Pharmacy.”

## LIMITATIONS

It is important to keep in mind the shortcomings and issues surrounding the impact factor. Coverage for tracking citations is entirely subject to those indexes that Clarivate Analytics has included. If a journal is not indexed in Web of Science, it will not be included in the scan for its references. For instance, *JAMA* is included in JCR, and so all the references of its appropriate articles are tracked. If a journal cites a *JAMA* article but that journal is not indexed, then that citation does not count toward *JAMA*’s impact factor or any other indicators.

Because it takes at least three years before an impact factor can be calculated, there is a tendency to favor older and larger journals. Such journals typically have more citations as they naturally have a larger body of previously published articles that are available to be cited.

Another problem is that self-citations (citations a publication makes to other articles published in the same journal) can bolster a journal’s impact factor [3]. JCR does have a way to address this, which is discussed in the “Major Features” section below.

Likewise, since review articles typically include so many more references, journals that heavily publish review articles can influence an impact factor as well.

## MAJOR FEATURES

While impact factors make up the backbone of JCR, there are several other metrics, indicators, and features provided throughout the resource that can aid in providing a fuller picture of a journal, article, or author’s influence.

- The **immediacy index** measures how frequently the average article from a journal is cited within the same year as its publication. This number could be useful for evaluating journals that publish cutting-edge research.
- The **eigenfactor score** is based on the number of times that articles from the journal that were published in the past five years have been cited in the JCR year, but it also considers which journals have contributed these citations so that highly cited journals will influence the network more than lesser cited journals. References from one article in a journal to another article from the same journal are removed so that eigenfactor scores are not influenced by journal self-citation.
- The **journal impact factor without self-cites** also addresses the criticism that impact factors can be easily influenced by self-citations. It is calculated exactly the same as the journal impact factor, but with one important exception: any citations to a publication that come from the publication itself (i.e., self-citations) are excluded from the numerator of the calculation.
- The **5-year journal impact factor** is the average number of times that articles from the journal that were published in the past five years have been cited in a given JCR year. This helps show the long-term citation trend for a journal.
- The **h-index** is usually used as a measure of scientific productivity and the scientific impact of an individual scientist, but it can also be used to rank journals.

Other features include being able to compare journals by trends and quartiles. There are a number of visualization charts and graphs that assist in seeing the relationship between citing and cited journals, which helps to trace a journal’s impact over time. Data from charts and graphs can be exported to portable document format (PDF), comma-separated value (CSV), and Excel (XLS) formats.

## AUDIENCE AND PURPOSE

JCR is a resource meant for researchers, librarians, clinicians, institutions, publishers, authors, and readers of medical, science, and social science literature to determine a journal’s influence in its relative field. There are many times when individuals or institutions will want to know a precise impact factor. Having access to the source means not having to rely on a publisher’s website for current and accurate data.

Knowing how best to use impact factors and journal rankings means acknowledging the limitations of solely relying on them to judge a journal’s scholarly worth. That said, impact factors and the journal’s ranking in its JCR category could be useful as part of a package including information such as databases where the journal is indexed. Those working in collection development and acquisitions would find JCR helpful when making purchasing decisions.

**Figure 2:**
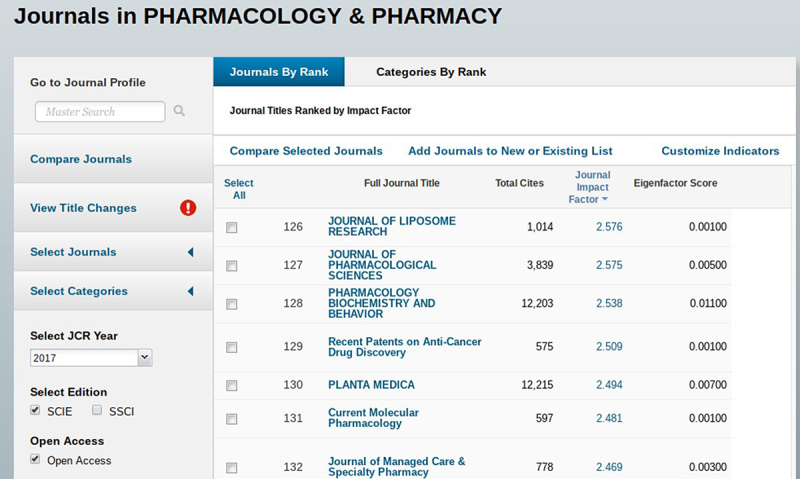
JCR list of journals in pharmacology and pharmacy

This reviewer has pulled lists from the category rankings to give authors other journals to consider for publishing. There is even an option to filter to open access journals, which is sometimes an author’s goal (Figure 2). Users can export their lists and include ranking, impact factor, and other indicators.

Anyone interested in tracking bibliometric citation patterns and trends will find the JCR information as well as their visualization charts and graphs very helpful.

## ACCESSIBILITY AND USABILITY

Access depends on having a license to the JCR resource. JCR lives on the Clarivate platform, allowing thorough integration with other Clarivate resources such as Web of Science and InCites; however, a subscription to one of these resources is necessary.

Searching and navigating JCR can be frustrating. It offers only basic title search functionality. A user has to input the title according to how Clarivate indexes that title for it to show up. The auto-fill is very sensitive and can miss a title, if care is not taken in typing out the journal title. Going from category data to journal rankings is clunky and nonintuitive. The hyperlink of the total number of journals must be used to get to the Journals By Rank.

## BRIEF COMPARISON TO OTHER SIMILAR PRODUCTS

There are several other journal metrics and ranking systems available. Perhaps the biggest competitor is SCImago journal rank indicator. It measures the scientific influence of scholarly journals based on both the number of citations received by a journal, very similar to how impact factor is collected (except they go back three years instead of two), and the importance or prestige of the journals where such citations come from [4].

The SCImago Journal & Country Rank is a publicly available portal, meaning there is no fee to access their information, which is a huge advantage over JCR. A limitation for this indicator is that it makes citation analyses based only on those journals that are indexed in Scopus. Likewise, JCR has a limitation of only being able to track citations of journals that are indexed in Web of Science. The difference is that Elsevier, the large publishing group, owns Scopus and could, therefore, potentially inflate the importance of its own journals.

## CONCLUSION

Purchasing JCR should be based on an institution’s research and publishing needs. JCR provides powerful and comprehensive bibliometric metrics from an objective source. While it has its limitations and newer metrics continually become available, JCR continues to be used globally.
